# Freely Behaving Mice Can Brake and Turn During Optogenetic Stimulation of the Mesencephalic Locomotor Region

**DOI:** 10.3389/fncir.2021.639900

**Published:** 2021-04-09

**Authors:** Cornelis Immanuel van der Zouwen, Joël Boutin, Maxime Fougère, Aurélie Flaive, Mélanie Vivancos, Alessandro Santuz, Turgay Akay, Philippe Sarret, Dimitri Ryczko

**Affiliations:** ^1^Département de pharmacologie-physiologie, Faculté de médecine et des sciences de la santé, Université de Sherbrooke, Sherbrooke, QC, Canada; ^2^Department of Medical Neuroscience, Atlantic Mobility Action Project, Brain Repair Center, Dalhousie University, Halifax, NS, Canada; ^3^Department of Training and Movement Sciences, Humboldt-Universität zu Berlin, Berlin, Germany; ^4^Berlin School of Movement Science, Humboldt-Universität zu Berlin, Berlin, Germany; ^5^Centre de recherche du Centre hospitalier universitaire de Sherbrooke, Sherbrooke, QC, Canada; ^6^Centre d’excellence en neurosciences de l’Université de Sherbrooke, Sherbrooke, QC, Canada; ^7^Institut de pharmacologie de Sherbrooke, Sherbrooke, QC, Canada

**Keywords:** locomotion, speed, braking, turning, mesencephalic locomotor region, cuneiform nucleus, Vglut2, optogenetics

## Abstract

A key function of the mesencephalic locomotor region (MLR) is to control the speed of forward symmetrical locomotor movements. However, the ability of freely moving mammals to integrate environmental cues to brake and turn during MLR stimulation is poorly documented. Here, we investigated whether freely behaving mice could brake or turn, based on environmental cues during MLR stimulation. We photostimulated the cuneiform nucleus (part of the MLR) in mice expressing channelrhodopsin in Vglut2-positive neurons in a Cre-dependent manner (Vglut2-ChR2-EYFP) using optogenetics. We detected locomotor movements using deep learning. We used patch-clamp recordings to validate the functional expression of channelrhodopsin and neuroanatomy to visualize the stimulation sites. In the linear corridor, gait diagram and limb kinematics were similar during spontaneous and optogenetic-evoked locomotion. In the open-field arena, optogenetic stimulation of the MLR evoked locomotion, and increasing laser power increased locomotor speed. Mice could brake and make sharp turns (~90°) when approaching a corner during MLR stimulation in the open-field arena. The speed during the turn was scaled with the speed before the turn, and with the turn angle. Patch-clamp recordings in Vglut2-ChR2-EYFP mice show that blue light evoked short-latency spiking in MLR neurons. Our results strengthen the idea that different brainstem neurons convey braking/turning and MLR speed commands in mammals. Our study also shows that Vglut2-positive neurons of the cuneiform nucleus are a relevant target to increase locomotor activity without impeding the ability to brake and turn when approaching obstacles, thus ensuring smooth and adaptable navigation. Our observations may have clinical relevance since cuneiform nucleus stimulation is increasingly considered to improve locomotion function in pathological states such as Parkinson’s disease, spinal cord injury, or stroke.

## Introduction

Coordination of speed, braking, and steering is essential to navigating the environment (Wynn et al., 2015). In the brainstem, the mesencephalic locomotor region (MLR) plays a key role in initiating and controlling locomotion (Shik et al., 1966; for review Ryczko and Dubuc, 2013). MLR glutamatergic neurons control locomotor speed from basal vertebrates to mammals (e.g., lamprey: Sirota et al., 2000; Brocard and Dubuc, 2003; Le Ray et al., 2003; salamanders: Cabelguen et al., 2003; mice: Lee et al., 2014; Roseberry et al., 2016; Capelli et al., 2017; Josset et al., 2018; Caggiano et al., 2018). A key function of the MLR is to elicit forward symmetrical locomotion by sending bilateral glutamatergic inputs to reticulospinal neurons that project to the spinal central pattern generator for locomotion (cat: Orlovski, 1970; lamprey: Buchanan and Grillner, 1987; Brocard et al., 2010; zebrafish: Kinkhabwala et al., 2011; Kimura et al., 2013; salamander: Ryczko et al., 2016a; mouse: Hägglund et al., 2010; Bretzner and Brownstone, 2013; Capelli et al., 2017; Lemieux and Bretzner, 2019; for review Grillner and El Manira, 2020). In mammals, the MLR sends descending projections to the gigantocellular nucleus (Gi), gigantocellular reticular nucleus, alpha part (GiA), gigantocellular reticular nucleus, ventral part (GiV), lateral paragigantocellular nucleus (LPGi), caudal raphe nuclei, intermediate reticular nucleus and medullary reticular nucleus, which all contain reticulospinal neurons (cat: Edwards, 1975; Steeves and Jordan, 1984; mouse: Bretzner and Brownstone, 2013; Capelli et al., 2017; Caggiano et al., 2018; for review Ryczko and Dubuc, 2013). Calcium imaging in isolated mouse brainstem indicated that Gi neurons expressing the marker Chx10, among which many are reticulospinal neurons, receive functional input from the MLR (Bretzner and Brownstone, 2013). *In vivo*, the photoinhibition of Gi neurons positive for the vesicular glutamatergic transporter 2 (Vglut2) disrupts ongoing locomotion (Lemieux and Bretzner, 2019). Glutamatergic reticulospinal neurons in the LPGi play an important role in relaying MLR speed commands in mice (Capelli et al., 2017).

Steering movements are induced by asymmetrical reticulospinal activity. Increased reticulospinal activity on one side induces ipsilateral steering movements in lamprey (Deliagina et al., 2000; Fagerstedt et al., 2001; Kozlov et al., 2014; Suzuki et al., 2019), zebrafish (Huang et al., 2013; Thiele et al., 2014), salamander (Ryczko et al., 2016c) and rat (Oueghlani et al., 2018). In mammals, steering commands are relayed by reticulospinal reticular neurons in the Gi that express the molecular marker Chx10. Their bilateral activation leads to a bilateral locomotor stop (Bouvier et al., 2015), while unilateral activation leads to a unilateral brake and turn (Cregg et al., 2020). A recent study based on viral injections in the spinal cord coupled with optogenetic stimulation in the brainstem uncovered that Gi Chx10-positive reticulospinal neurons projecting to the lumbar spinal cord decrease locomotor speed, whereas those projecting to the cervical spinal cord evoke the ipsilateral head movement preceding turning (Usseglio et al., 2020). Gi Chx10-positive neurons receive a major glutamatergic input from the contralateral superior colliculus (SC; Cregg et al., 2020; Usseglio et al., 2020), a region involved in visuomotor transformations (Felsen and Mainen, 2008; Shang et al., 2015; Zingg et al., 2017; for review Oliveira and Yonehara, 2018).

The interactions between brainstem substrates controlling speed and those controlling braking and turning are unknown. Whether braking or turning can be done during MLR stimulation is poorly documented in mammals. In mice, MLR-evoked locomotion was recorded on a trackball (Lee et al., 2014; Roseberry et al., 2016), on a treadmill (Josset et al., 2018), in a linear corridor (Josset et al., 2018; Caggiano et al., 2018), or in a hole board test (Caggiano et al., 2018), but the direction of motion was not measured in these studies. Capelli et al. (2017) carried out a locomotion directional analysis in the open field in response to stimulation of Vglut2-positive neurons of the LPGi, but not in response to MLR stimulation. In decerebrated cats held over a treadmill oriented in various directions, MLR stimulation generated well-coordinated locomotion only when the treadmill was going in the front-to-rear direction, suggesting that the MLR could only generate forward motion (Musienko et al., 2012).

Here, we examined whether freely behaving mice can brake or turn by integrating environmental cues during optogenetic stimulation of the MLR. In mice expressing channelrhodopsin in neurons positive for the Vglut2 (Vglut2-ChR2-EYFP mice), we targeted the cuneiform nucleus (CnF), the MLR subregion that controls the largest range of speeds (Josset et al., 2018; Caggiano et al., 2018). We used deep learning to detect locomotor movements in a linear corridor and in an open-field arena. It is relevant to determine whether MLR-evoked locomotion can be dynamically adapted to the environment, as MLR stimulation is explored to improve locomotor function in Parkinson’s disease (Plaha and Gill, 2005; Hamani et al., 2016a, b; Goetz et al., 2019) and in animal models of spinal cord injury (Bachmann et al., 2013; Richardson, 2014; Roussel et al., 2019; for review Chari et al., 2017) and stroke (Fluri et al., 2017). We focused on the CnF, which is increasingly considered as the optimal subregion to target within the MLR (Chang et al., 2020).

## Materials and Methods

The procedures were as per the guidelines of the Canadian Council on Animal Care and approved by the animal care and use committees of the Université de Sherbrooke.

### Animals

**Figure 1:**
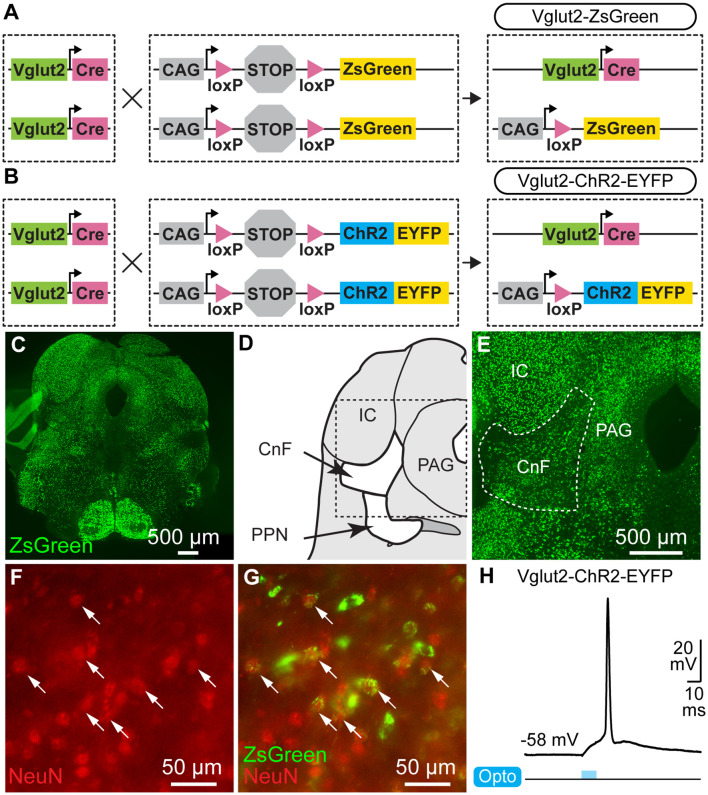
Cre-dependent expression of channelrhodopsin or ZsGreen in neurons of the Mesencephalic Locomotor Region (MLR) expressing the vesicular glutamate transporter 2 (Vglut2). **(A)** For anatomical experiments, homozygous mice expressing Cre-recombinase under the control of the Vglut2 promoter (Vglut2-Cre, see “Materials and Methods” section) were crossed with homozygous mice with ZsGreen preceded by a STOP cassette flanked by loxP sites preventing ZsGreen expression. In the resulting heterozygous mice (Vglut2-ZsGreen), if Vglut2 is expressed during cell lifetime, Cre-dependent recombination removes the STOP cassette, allowing permanent expression of ZsGreen under control of the CAG promoter. **(B)** For optogenetic experiments, mice homozygous for Vglut2-Cre were crossed with mice homozygous for channelrhodopsin (ChR2) and enhanced yellow fluorescent protein (EYFP) preceded by a STOP cassette flanked by loxP sites preventing their expression. In the resulting heterozygous mice (Vglut2-ChR2-EYFP), if Vglut2 is expressed during cell lifetime, Cre-dependent recombination removes the STOP cassette, allowing permanent expression of ChR2-EYFP under control of the CAG promoter. **(C)** Photomicrographs of transversal brain slices from Vglut2-ZsGreen mice at the MLR level. **(D)** Schematic representation of a brain slice at the MLR level. **(E)** Higher magnification of the brain slice in **(C)** at the level of the CnF. **(F,G)** Epifluorescent images taken in the CnF, showing that cells expressing ZsGreen (green) are immunopositive for the neuronal marker NeuN (red). **(H)** Whole-cell patch-clamp recording of a neuron recorded in a brainstem slice of a Vglut2-ChR2-EYFP mouse at the level of the MLR. The neuron spikes an action potential at short latency in response to a 10 ms blue light pulse. CnF, cuneiform nucleus; IC, inferior colliculus; PAG, periaqueductal gray; PPN, pedunculopontine nucleus.

Ten mice were used. We used Vglut2-Cre mice [Jackson laboratories, #028863, Vglut2-ires-cre knock-in (C57BL/6J); Vong et al., 2011; Figures 1A,B], ChR2-EYFP-lox mice (Ai32 mice, Jackson laboratory, #024109, B6.Cg-Gt(ROSA)26Sor^tm32(CAG-COP4*H134R/EYFP)Hze^/J; Madisen et al., 2012; Figure 1B), and ZsGreen-lox mice (Ai6 mice, Jackson laboratory, #007906, B6.Cg-*Gt(ROSA)26Sor^tm6(CAG-ZsGreen1)Hze^*/J; Madisen et al., 2012; Figure 1A). We crossed homozygous Vglut2-Cre mice with homozygous ChR2-EYFP-lox mice to obtain the double heterozygous Vglut2-ChR2-EYFP mice. We crossed homozygous Vglut2-Cre mice with homozygous ZsGreen-lox mice to obtain the double heterozygous Vglut2-ZsGreen mice. Animals had *ad libitum* access to food and water, with lights on from 6 AM to 8 PM. Mice were 16–36 weeks old for *in vivo* optogenetics (three males, two females), 10–18 weeks old for neuroanatomy (one male, two females), and 15–23 days old for patch-clamp experiments (one male, one undetermined).

### Genotyping

Mice were genotyped as previously described (Fougère et al., 2020). Briefly, DNA was extracted from ear punches using the Taq DNA polymerase (NEB, Ipswich, MA, USA). Genotyping was performed using the primers recommended by the supplier (Jackson laboratory). Vglut2-ires-Cre mice were genotyped using mixed primer PCR employing Vglut2-ires-Cre-Com-F (AAGAAGGTGCGCAAGACG), Vglut2-ires-Cre-Wt-R (CTGCCACAGATTGCACTTGA), and Vglut2-ires-Cre-Mut-R (ACACCGGCCTTATTCCAAG). Amplification of wild-type genomic DNA yielded a 245 bp PCR product whereas amplification from the mutant locus yielded a 124 bp PCR product, as expected according to the supplier. ChR2-lox mice and ZsGreen-lox mice were genotyped using mixed primer PCR employing ZsGreen-ChR2-lox-Wt-F (AAGGGAGCTGCAGTGGAG TA), ZsGreen-ChR2-lox-Wt-R (CCGAAAATCTGTGGGAAGTC), ZsGreen-ChR2-lox-Mut-R (GGCATTAAAGCAGCGTATCC), and either ChR2-lox-Mut-F (ACATGGTCCTGCTGGAGTTC) or ZsGreen-lox-Mut-F (AACCAGAAGTGGCACCTGAC). Amplification of wild-type genomic DNA yielded a 297 bp PCR product whereas amplification from the mutant ChR2-lox locus yielded a 212 bp PCR product and amplification of the mutant ZsGreen-lox locus yielded a 199 bp PCR product, as expected according to the supplier.

### Optical Fiber Implantation

Mice were anesthetized using isoflurane (induction: 5%, 500 ml/min; maintenance: 1.5–2.5%, 100 ml/min) delivered by a SomnoSuite (Kent Scientific, Torrington, CT, USA). Mice were placed in a Robot Stereotaxic instrument coupled with StereoDrive software (Neurostar, Tübingen, Germany) to perform unilateral implantation of an optical fiber (200 μm core, 0.22 NA, Thorlabs, Newton, NJ, USA) held in a 5 mm ceramic or stainless-steel ferrule 500 μm above the right CnF at −4.80 mm anteroposterior, +1.10 mm mediolateral, −2.40 mm dorsoventral relative to bregma (Josset et al., 2018; Caggiano et al., 2018). The ferrule was secured on the cranium using two 00-96 × 1/16 mounting screws (HRS Scientific, QC, Canada) and dental cement (A-M Systems, Sequim, WA, USA).

### *In vivo* Optogenetic Stimulation

The optical fiber was connected using a pigtail rotary joint (Thorlabs) to a 470 nm laser (Ikecool, Anaheim, CA, USA) or a 589 nm laser (Laserglow, ON, Canada) driven by a Grass S88X that generated the stimulation trains (10 s train, 10 ms pulses, 20 Hz; Caggiano et al., 2018; Josset et al., 2018). To visualize optogenetic stimulation, a small (diameter 0.5 cm), low-power (0.13 W) red LED that received a copy of the stimulation trains was placed in the field of view of the camera placed above the open field. The 470 nm light source was adjusted to 6–27% of laser power and the 589 nm to 40–53% of laser power. The corresponding power measured at the fiber tip with a power meter (PM100USB, Thorlabs) was 0.1–16.0 mW for the 470 nm laser and 1.7–9.4 mW for the 589 nm laser.

### Open-Field Locomotion

Locomotor activity was filmed from above in a 40 × 40 cm arena at 30 fps using a Logitech Brio camera coupled to a computer equipped with ANY-maze software (Stoelting Co., Wood Dale, IL, USA) or using a Canon Vixia HF R800 camera. Locomotor activity was recorded during trials of 15 min during which 10 stimulation trains were delivered every 80 s at various laser powers. Video recordings were analyzed on a computer equipped with DeepLabCut (version 2.1.5.2), a software-based on deep learning to track user-defined body parts (Mathis et al., 2018; Nath et al., 2019), and a custom Matlab script (Mathworks, Natick, MA, USA). We tracked frame by frame the body center position, the corners of the arena for distance calibration, and the low-power LED to detect optogenetic stimulations. Timestamps were extracted using Video Frame Time Stamps (Matlab File Exchange). Body center positions and timestamps were used to calculate locomotor speed in cm/s. To compare and average speed over time for different stimulations, the data were downsampled to 20 Hz. Body center positions were excluded if their likelihood of detection by DeepLabCut was <0.8, if they were outside of the open-field area, or if body center speed exceeded the maximum locomotor speed recorded in mice (334 cm/s, Garland et al., 1995).

For offline analysis of turning movements in the arena’s corners, we defined regions of interest (ROIs) as circles (radius 20 cm) centered on each corner. The turning point was defined as the intersection of the mouse’s trajectory with the bisector of each corner (i.e., diagonal of the corner; Figure 5D). The coordinates of the turning point were calculated using Curve intersections (Matlab File Exchange). Within an ROI, a turn was defined as a trajectory that started at least 5 cm away from the turning point, crossed the diagonal, and ended at least 5 cm away from the turning point. Turns were excluded if the mouse crossed the diagonal more than once without leaving the ROI. The turn angle was measured between the first point of the trajectory, the turning point, and the last point of the trajectory.

**Figure 2:**
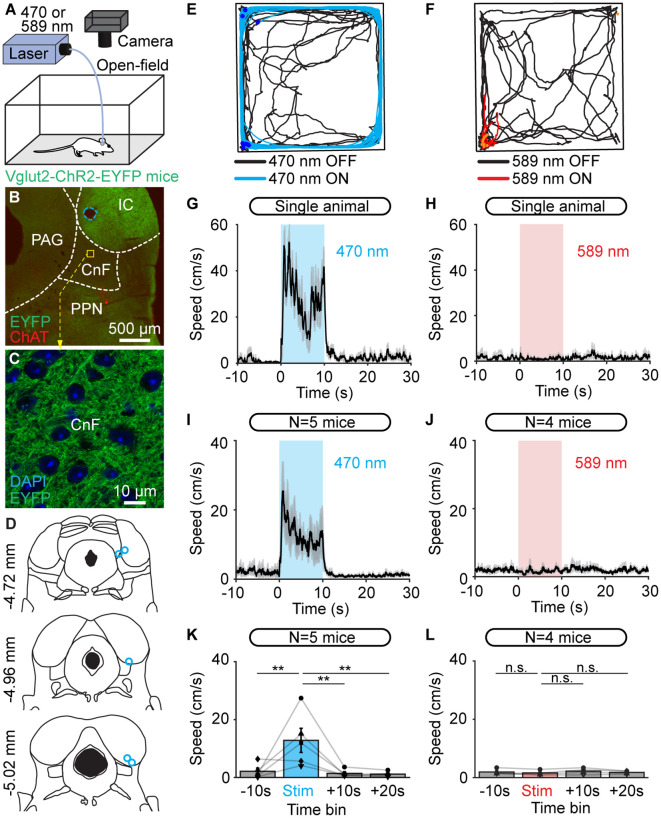
Optogenetic stimulation of the cuneiform nucleus (CnF) increases locomotor speed in the open-field arena in Vglut2-ChR2-EYFP mice. **(A)** An optic fiber implanted in the right CnF was connected to a blue laser (470 nm) or red laser (589 nm). Animals were placed in an open-field arena (40 × 40 cm) and their movements were recorded using a camera placed above. **(B)** Photomicrograph showing the position of the optic fiber (dashed blue line) ~500 μm above the target site. The cholinergic neurons of the pedunculopontine nucleus (PPN; choline acetyltransferase positive, ChAT, red) and the expression of EYFP (green) are visible. **(C)** Magnification of the slice in **(B)** at the level of the CnF, showing the expression of ChR2-EYFP. **(D)** Location of the optic fibers (blue circles) after histological verification as illustrated in **(B)**, with the relative position to the bregma. **(E,F)** Raw data showing the effects of 10 optogenetic stimulations with a 470 nm laser (**E**, light blue lines, 10 s train, 20 Hz, 10 ms pulses, 11% of laser power) or a 589 nm laser (**F**, red lines, 10 s train, 20 Hz, 10 ms pulses, 53% of laser power). A time interval of 80 s was left between two trains of stimulation. Therefore, the time elapsed is different during stimulation (10 s, red or blue traces) and rest (80 s, black traces). The position of the animal’s body center was tracked frame by frame with DeepLabCut (see “Materials and Methods” section). Dark blue dots **(E)** and orange dots **(F)** illustrate the onset of each stimulation. **(G,H)** Locomotor speed (mean ± SEM) as a function of time before, during, and after a 10 s optogenetic stimulation (onset at *t* = 0 s) with a 470 nm laser **(G)** or 589 nm laser **(H)** in a single animal (same animal as in **E,F**). **(I,J)** Locomotor speed (mean ± SEM) before during and after optogenetic stimulation with a 470 nm laser in five animals (**I**, 10 stimulations per animal, 10–24% of laser power) and with the 589 nm laser in four animals (**J**, 10 stimulations per animal, 40–53% of laser power). **(K,L)** Locomotor speed (mean ± SEM) before (−10 to 0 s), during (0 to +10 s), and after optogenetic stimulation ( +10 to +20 s and +20 to +30 s) with the 470 nm laser in five animals **(K)** and with the 589 nm laser in four animals (**L**; 10 stimulations per animal, **P < 0.01, n.s.: not significant, *P* > 0.05, Student-Newman-Keuls test after a one way ANOVA for repeated measures, *P* < 0.01 in **K** and *P* > 0.05 in **L**).

**Figure 3:**
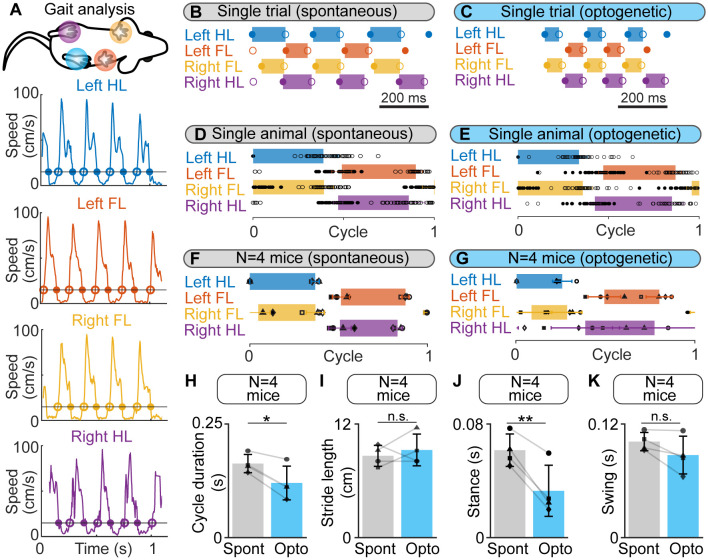
Gait diagrams during spontaneous locomotion and locomotion evoked by optogenetic stimulation of the cuneiform nucleus (CnF) in Vglut2-ChR2-EYFP mice in a linear corridor. **(A)** Mouse forelimbs (FL) and hindlimbs (HL) were filmed from below at 300 fps in a transparent linear corridor and the position of each limb was tracked frame by frame with DeepLabCut (see “Materials and Methods” section). The four panels show the movement speed of each paw as a function of time. Cycle duration was defined as the time duration between two touchdowns of the left hindlimb (HL) using a speed threshold of 15 cm/s to define the transitions between swing and stance phases. Full circles are touchdowns; empty circles are lift-offs. **(B,C)** Gait diagram for each limb obtained during a single spontaneous locomotor bout **(B)** and a locomotor bout evoked by optogenetic stimulation in the same animal (470 nm laser, 10 s train, 20 Hz, 10 ms pulses, 8% of laser power). Stance phase duration was drawn with a rectangle only when both touchdown and lift-off were present in the recording. **(D,E)** Gait diagrams during a normalized locomotor cycle, showing the stance phase start (mean ± SD) and end (mean ± SD) during 16 spontaneous locomotor bouts (45 steps) and during 8 locomotor bouts evoked by optogenetic stimulation in the same animal (48 steps, same stimulation parameters as in **E**). The cycle has been normalized to the left HL’s touchdown. **(F,G)** Normalized gait diagram showing the touchdown (mean ± SD) and lift-off (mean ± SD) pooled from four mice during a total of 55 spontaneous locomotor bouts (8–16 trials per animal, 13–45 steps per animal) and from four mice during a total of 30 locomotor bouts (6–8 bouts per animal, 8–48 steps per animal) evoked by CnF optogenetic stimulation (470 nm laser, 10 s train, 20 Hz, 10 ms pulses, 8–15% of laser power). The data from each animal are illustrated with a different symbol. **(H–K)** Comparison of cycle duration **(H)** stride length **(I)** stance duration **(J)** and swing duration **(K)** in four animals during spontaneous optogenetic-evoked locomotion (same animals as in **F,G**). *P < 0.05, **P < 0.01, n.s., not significant, *P* > 0.05, paired *t*-tests.

**Figure 4:**
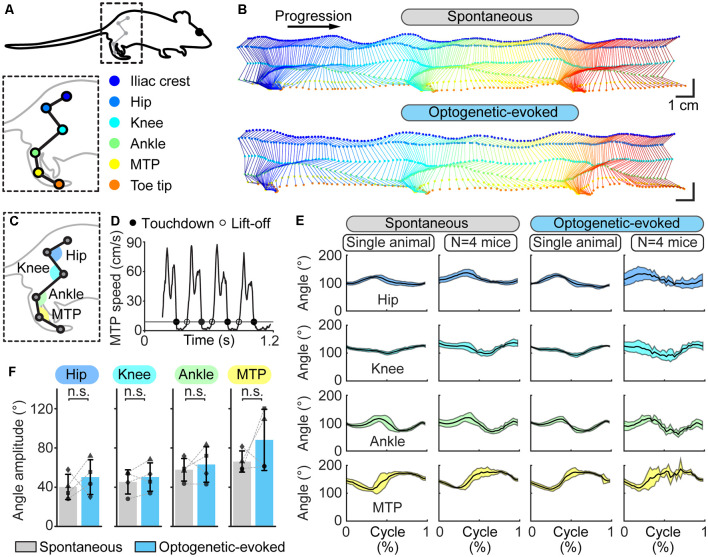
Hindlimb kinematics during spontaneous locomotion and locomotion evoked by optogenetic stimulation of the cuneiform nucleus (CnF) in Vglut2-ChR2-EYFP mice in a linear corridor. **(A)** Six hindlimb joints were labeled with a white paint marker and were filmed from the side at 300 fps in a transparent linear corridor and the trajectory of each joint was extracted with DeepLabCut and plotted in a different color (see “Materials and Methods” section). **(B)** Side view of the hindlimb joints during spontaneous locomotion (top) and optogenetic-evoked locomotion (470 nm laser, 10 s train, 20 Hz, 10 ms pulses, 8% of laser power) (bottom). Total time elapsed from first to last frames is 700 ms (top) and 500 ms (bottom). **(C)** Joint angles at the hip, knee, ankle, and metatarsophalangeal joint (MTP) levels were calculated frame by frame using the position of the joint of interest and those of two proximal joints. **(D)** Cycle duration was defined as the time duration between two consecutive touchdowns of the MTP using a speed threshold of 9 cm/s to define the transitions between swing and stance phases. Full circles are touchdowns; empty circles are lift-offs. **(E)** Joint angles (mean ± SD) at the hip, knee, ankle and MTP levels plotted for a normalized locomotor cycle during spontaneous locomotion (29 steps) and locomotion evoked by optogenetic stimulation (31 steps; 470 nm laser, 10 s train, 20 Hz, 10 ms pulses, 8% of laser power). For the pooled data, joint angles (mean ± SD) of four animals plotted for a normalized locomotor cycle during spontaneous locomotion (8–29 steps per animal) and locomotion evoked by optogenetic stimulation are shown (2–31 steps per animal; 470 nm laser, 10 s train, 20 Hz, 10 ms pulses, 8–15% of laser power). **(F)** Amplitude of the hip, knee, ankle, and MTP angles measured in four animals during spontaneous and optogenetic evoked locomotion (same data as in **E**). n.s., not significant, *P* > 0.05, paired *t*-tests.

**Figure 5:**
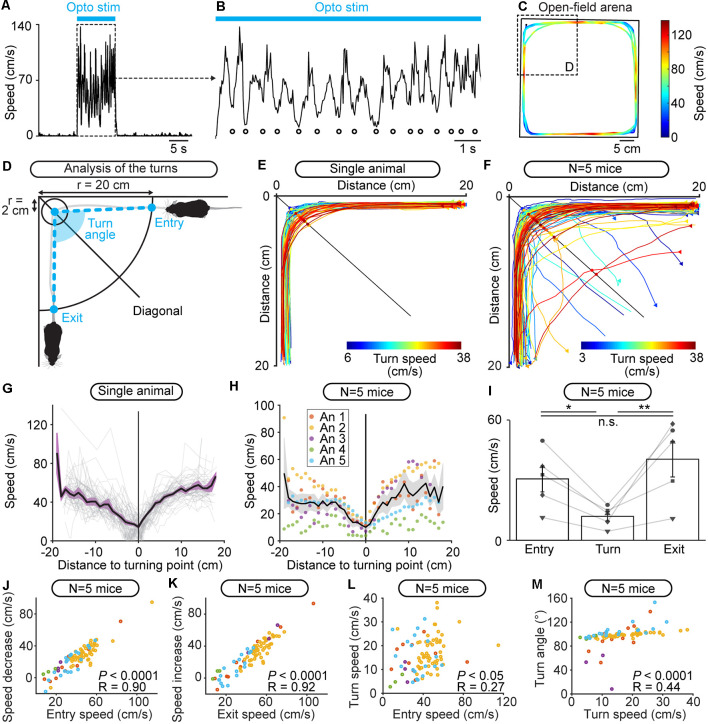
Freely behaving mice brake and turn during locomotion evoked by optogenetic stimulation of the cuneiform nucleus (CnF) in Vglut2-ChR2-EYFP mice in the open-field arena. **(A)** Raw data showing that locomotor speed is modulated during optogenetic stimulation of the CnF (470 nm laser, 10 s train, 20 Hz, 10 ms pulses, 11% of laser power). **(B)** Magnification of **(A)** showing rhythmic speed decrease (white circles) during CnF stimulation (blue solid line). **(C)** Color plot illustrating the locations of the speed decreases during CnF stimulation in the open-field arena (same data as in **A,B**). Colder colors (blue) illustrate slower speeds. **(D)** Animal’s speed was measured when moving in 20 cm circles centered on each corner of the arena. The speed at the entry of the corner, during the turn (in a 2 cm circle centered on the location where the animal crossed the corner’s diagonal), and at the exit of the corner was calculated (see “Materials and Methods” section). The turn angle was measured between the positions of the animal: (i) at the entry of the corner; (ii) during the turn; and (iii) when exiting the corner (see “Materials and Methods” section). **(E,F)** Raw data showing the extracted locomotor trajectories in the corners of the arena during optogenetic-evoked locomotion in a single animal (E, 470 nm laser, 10 s train, 20 Hz, 10 ms pulses, 11% of laser power) and in five animals (**F**, 470 nm laser, 10 s train, 20 Hz, 10 ms pulses, 10–24% of laser power). Warmer colors (red) illustrate trajectories with higher speeds during the turn. Triangles illustrate movement onsets. Dots illustrate diagonal crossings. **(G)** Locomotor speed as a function of the distance to the corner’s diagonal during each turn (gray) shown in **(E)** for a single animal. In black, the averaged speed (± sem in purple) is shown. **(H)** Locomotor speed as a function of the distance to the diagonal during the turns of the five animals (An) illustrated in **(F)**. In black, the mean speed (± sem in gray) is shown. **(I)** Entry speed, turn speed and exit speed for five animals (10 stimulations per animal, *P < 0.05, **P < 0.01, n.s., not significant, *P* > 0.05, Student-Newman-Keuls test after a one way ANOVA for repeated measures, *P* < 0.01). **(J)** Relationship between the speed at corner entry, and the difference between entry speed and turn speed in five mice (linear fit, *P* < 0.0001, *R* = 0.90, *n* = 87 turns pooled from 50 stimulations, 10 stimulations per animal). **(K)** Relationship between the speed at corner exit, and the difference between exit speed and turn speed (linear fit, *P* < 0.0001, *R* = 0.92). **(L)** Relationship between the speed at corner entry and the turn speed (linear fit, *P* < 0.05, *R* = 0.27). **(M)** Relationship between the turn speed and the turn angle (linear fit, *P* < 0.0001, *R* = 0.44).

Locomotor speed during the start of the turn (“entry speed”), around the turning point (“turn speed”), and during the end of the turn (“exit speed”) were measured using the distance of each point of the trajectory to the turning point. These distance values were binned (width: 1 cm) and speed values were averaged per bin. Entry speed was averaged from the four most distal distance bins before the turning point and at least 5 cm away from the turning zone (2 cm radius around the turning point). Turn speed was averaged from the four distance bins located within the turning zone. Exit speed was averaged from the four most distal distance bins after the turning point and at least 5 cm away from the turning zone. Turns were removed from the analysis if fewer than four bins were available to calculate entry, turn, or exit speed.

### Footfall Patterns and Limb Kinematics

To label hindlimb joints for offline tracking with DeepLabCut, mice were anesthetized with isoflurane (induction: 5%, 500 ml/min; maintenance: 1.5–2.5%, 100 ml/min), the hindlimb was shaved, and white dots (diameter ~2 mm) were drawn on the iliac crest, hip, knee, ankle, and metatarsophalangeal (MTP) joints, and toe tip using a fine-tip, oil-based paint marker (Sharpie). For footfall pattern tracking, no labeling of paw underside was needed. Animals recovered for 20 min after anesthesia and were placed in a 1 m long, 8 cm wide transparent corridor. The footfall pattern and hindlimb kinematics were recorded at 300 fps using two high-speed Genie Nano Camera M800 cameras (Teledyne DALSA, Waterloo, ON, Canada) coupled to a computer equipped with Norpix Streampix software (1st Vision, Andover, MA, USA). Hindlimb kinematics were recorded with a camera placed on the side of the corridor. Footfall patterns were recorded with a camera placed on the side and directed toward a 45-degree mirror placed below the corridor. For distance calibration, four markers (diameter 0.5 cm) were distributed 5 cm apart and placed in the field of view of each camera. To detect optogenetic stimulation, a low-power LED that received a copy of the stimulation trains was placed in the field of view of both cameras. Animals were recorded during spontaneous locomotion evoked by a gentle touch of the animal’s tail and during optogenetic-evoked locomotion.

For the footfall pattern, videos recorded from below were used to track the position of the MTPs of the four paws with DeepLabCut (Mathis et al., 2018; Nath et al., 2019; Santuz and Akay, 2020). Paw speeds were calculated and smoothened with a moving average (on five frames) using a custom Matlab script. Touchdown and lift-off were defined for each paw as the time points at which each MTP speed respectively fell below or rose above 15 cm/s. The touchdown and lift-off time points of each limb were identified using Curve intersections (Matlab File Exchange) and were normalized to the step cycle of the left hindlimb to generate normalized gait diagrams (Josset et al., 2018). A step cycle was defined as the time between two consecutive touchdowns of the left hindlimb (Caggiano et al., 2018). Cycle duration, stance phase duration, swing phase duration, and stride length were calculated (Caggiano et al., 2018).

For hindlimb kinematics, the positions of the joints and toe tip were detected using DeepLabCut. The moving average of the MTP speed was used to determine the stance and swing phases by detecting the touchdown and lift-off times with a speed threshold of 9 cm/s, and a minimum of eight frames above the threshold for the lift-off detection. The joint positions were used to extract the angles of the hip, knee, and ankle joints (Figure 4C). The angular variations as a function of time were normalized to step cycle duration using MTP touchdown times as a reference (Leblond et al., 2003).

Frames were excluded from the analysis if the MTPs of any paw (for the footfall pattern) or any limb joints or the toe tip (for limb kinematics) had a likelihood of detection <0.8 by DeepLabCut. Frames were excluded from the analysis if any paw’s or joint’s speed exceeded 400 cm/s, i.e., the maximum locomotor speed of a mouse with a 20% margin to account for increased speed of individual body parts (Garland et al., 1995). Frames were excluded from the analysis if the distance between two adjacent joints was greater than 2.3 cm, i.e., the mean length of the tibia in wildtype mice with a 30% margin to account for individual variation between mice (Kamal et al., 2015).

### DeepLabCut Networks

For open-field locomotion analysis, we labeled six landmarks on 520 frames taken from 20 videos of eight different animals assigning 95% of those images to the training set without cropping. The landmarks were the body center, the four corners of the arena, and the low-power LED to visualize optogenetic stimulation. We used a ResNet-50-based neural network (He et al., 2016; Insafutdinov et al., 2016) with default parameters for 1,030,000 training iterations. We validated with one shuffle and found that the test error was 2.28 pixels and the train error 1.85 pixels.

For footfall pattern analysis, we labeled nine landmarks 489 frames taken from 27 videos of nine animals assigning 95% of those images to the training set without cropping. The landmarks were the four paws, the four distance calibration markers, and the low-power LED to visualize optogenetic stimulation. We used a ResNet-50-based neural network (He et al., 2016; Insafutdinov et al., 2016) with default parameters for 1,030,000 training iterations. We validated with one shuffle and found that the test error was 2.31 pixels and the train error 1.76 pixels.

For limb kinematics analysis, we labeled 11 landmarks on 906 frames taken from 44 videos of seven different animals assigning 95% of those images to the training set without cropping. The landmarks were the five joints and the toe tip, the four distance calibration markers, and the low-power LED to visualize optogenetic stimulation. We used a ResNet-50-based neural network (He et al., 2016; Insafutdinov et al., 2016) with default parameters for 1,030,000 training iterations and one refinement of 1,030,000 iterations. We validated with one shuffle and found that the test error was 2.03 pixels and the train error 1.87 pixels.

### Patch-Clamp Recordings

Coronal brainstem slices were obtained from 15-to 23-days old mice as previously described (Ryczko et al., 2020a). Briefly, mice were anesthetized with isoflurane (0.5–1 ml of isoflurane in a 1.5 L induction chamber) and decapitated with a guillotine. The cranium was opened and the brain removed to be dipped in an ice-cold sucrose-based solution (in mM: 3 KCl, 1.25 KH_2_PO_4_, 4 MgSO_4_, 26 NaHCO_3_, 10 Dextrose, 0.2 CaCl_2_, 219 Sucrose, pH 7.3–7.4, 300–320 mOsmol/kg) bubbled with 95% O_2_ and 5% CO_2_. MLR slices (350 μm thick) were prepared with a VT1000S vibrating blade microtome (Leica Microsystems, Concord, ON, Canada) and stored at room temperature for 1 h in artificial cerebrospinal fluid (aCSF; in mM: 124 NaCl, 3 KCl, 1.25 KH_2_PO_4_, 1.3 MgSO_4_, 26 NaHCO_3_, 10 Dextrose, and 1.2 CaCl_2_, pH 7.3–7.4, 290–300 mOsmol/kg) bubbled with 95% O_2_ and 5% CO_2_. Whole-cell patch-clamp recordings were done in a chamber perfused with bubbled aCSF under an Axio Examiner Z1 epifluorescent microscope (Zeiss, Toronto, ON, Canada), differential interference contrast (DIC) components, and an ORCA-Flash 4.0 Digital CMOS Camera V3 (Hamamatsu Photonics, Hamamatsu, Japan). Patch pipettes were pulled from borosilicate glass capillaries (1.0 mm outside diameter, 0.58 mm inside diameter; 1B100F-4, World Precision Instruments, FL, USA) using a P-1000 puller (Sutter Instruments). Pipettes with a resistance of 6–12 MΩ were filled with a solution containing (in mM) 140 K-gluconate, 5 NaCl, 2 MgCl_2_, 10 HEPES, 0.5 EGTA, 2 Tris ATP salt, 0.4 Tris GTP salt, pH 7.2–7.3, 280–300 mOsmol/kg, 0.05 Alexa Fluor 594 or 488, and 0.2% biocytin). Positive pressure was applied through the glass pipette and neurons were approached using a motorized micromanipulator (Sutter instruments). A gigaseal was established and the membrane potential was held at −60 mV. Liquid junction potential was not compensated. The membrane patch was suctioned, and the pipette resistance and capacitance were compensated electronically. Neurons were discarded when action potentials were less than 40 mV or when the resting membrane potential was too depolarized (>−45 mV). Patch-clamp signals were acquired with a Multiclamp 700B coupled to a Digidata 1550B and a computer equipped with PClamp 10 software (Molecular Devices, Sunnyvale, CA, USA). Optogenetic stimulations (475 nm, 10 ms pulses, 2.5–5% of LED power) were applied using the 475 nm LED of a Colibri 7 illumination system (Zeiss).

### Histology and Immunofluorescence

Procedures were as previously reported (Fougère et al., 2020). Briefly, mice were anesthetized using isoflurane (5%, 2.5 L per minute) and transcardially perfused with 50 ml of a phosphate buffer solution (0.1 M) containing 0.9% NaCl (PBS, pH = 7.4), followed by 40–75 ml of a PBS solution containing 4% (wt/vol) of paraformaldehyde (PFA 4%). Post-fixation of the brains was performed in a solution of PFA 4% for 24 h at 4°C. Then, the brains were incubated in a PBS solution containing 20% (wt/vol) sucrose for 24 h before histology. Brains were snap-frozen in methylbutane (−45°C ± 5°C) and sectioned at −20°C in 40 μm-thick coronal slices using a cryostat (Leica CM 1860 UV). Floating sections of the MLR were collected under a Stemi 305 stereomicroscope (Zeiss) and identified using the atlas of Franklin and Paxinos (2008).

For immunofluorescence experiments, all steps were carried out at room temperature unless stated otherwise. The sections were rinsed in PBS for 10 min three times and incubated for 1 h in a blocking solution containing 5% (vol/vol) of normal donkey serum and 0.3% Triton X-100 in PBS. The sections were then incubated at 4°C for 48 h in a PBS solution containing the primary antibody against choline acetyltransferase [ChAT; goat anti-choline acetyltransferase, Sigma AB144P, lot 3018862 (1:100), RRID: AB_2079751] or the neuronal marker NeuN [rabbit anti-NeuN, Abcam AB177487, lot GR3250076–6 (1:1,000), RRID: AB_2532109] and agitated with an orbital shaker. The sections were washed three times in PBS and incubated for 4 h in a solution containing the appropriate secondary antibody to reveal ChAT [donkey anti-goat Alexa 594, Invitrogen A11058, lot 1975275 (1:400), RRID: AB_2534105] or NeuN [with a donkey anti-rabbit Alexa Fluor 594, Invitrogen A21207 lot 1890862 (1:400), RRID: AB_141637; or a donkey anti-rabbit Alexa Fluor 647, ThermoFisher A31573 lot 2083195 (1:400), RRID: AB_2536183]. The slices were rinsed three times in PBS for 10 min and mounted on Colorfrost Plus glass slides (Fisher) with a medium with DAPI (Vectashield H-1200) or without DAPI (Vectashield H-1000), covered with a 1.5 type glass coverslip and stored at 4°C before observation. Brain sections were observed using a Zeiss AxioImager M2 microscope equipped with StereoInvestigator 2018 software (v1.1, MBF Bioscience). Composite images were assembled using StereoInvestigator. The levels were uniformly adjusted in Photoshop CS6 (Adobe) to make all fluorophores visible and avoid pixel saturation, and digital images were merged.

### Specificity of the Antibodies

The AB177487 anti-NeuN has been widely used to label the neuronal marker NeuN (also called Fox-3, see Mullen et al., 1992; Kim et al., 2009) in mouse brain tissues by us (Fougère et al., 2020) and others (Saito et al., 2018; Joy et al., 2019). According to the supplier, this monoclonal purified antibody (clone EPR12763) is directed towards a synthetic peptide of the residues 1–100 of the human NeuN. NeuN is present in most mouse neurons, but not in cerebellar Purkinje cells, olfactory bulb mitral cells, and retinal photoreceptor cells (Mullen et al., 1992). According to the supplier, AB177487 labels NeuN in HeLa cell lysates and in the brains of mice, rats, and humans. It detects two bands at 45–50 kDA in Western blots performed on the mouse, rat, or human brain tissues.

The AB144P ChAT antibody has been widely used to label cholinergic neurons in lamprey (Pombal et al., 2001; Le Ray et al., 2003; Quinlan and Buchanan, 2008; Ryczko et al., 2013), salamander (Marín et al., 1997; Cabelguen et al., 2003; Ryczko et al., 2016a), rat (Ryczko et al., 2016b), human (Massouh et al., 2008; Ryczko et al., 2016b) and mouse brain tissues (Steinkellner et al., 2019). This affinity-purified polyclonal antibody is raised against the human placental enzyme. The supplier has tested its specificity in human placenta lysates and using western blots on mouse brain lysates, where it detects a band of 68–70 kDA. It labels neurons expressing a fluorescent protein under the control of the ChAT promoter in mice (Bloem et al., 2014). In all cases, removing the primary antibodies resulted in the absence of labeling on brain sections.

### Specificity of the Transgenic Mice

#### Vglut2-ires-Cre Mouse

These mice are widely used to express Cre-recombinase in glutamatergic Vglut2-positive neurons without interfering with *Vglut2* gene expression (Vong et al., 2011). When Vglut2-ires-Cre are crossed with a lox-GFP mouse, GFP-positive neurons are found in glutamatergic regions (positive for *Vglut2* mRNA) and absent from GABAergic regions (positive for the vesicular GABA transporter mRNA; Vong et al., 2011). When Vglut2-ires-Cre are crossed with a lox-tdTomato mouse, the cells labeled in the dorsal horn of the spinal cord are immuno-positive for NeuN and immuno-negative for Pax2 and Wilm’s tumor 1, two markers of inhibitory neurons (Haque et al., 2018; Wang et al., 2018). Chemogenetic activation of Vglut2-Cre neurons increases the frequency of synaptic excitatory currents recorded with patch-clamp in spinal cord slices (Wang et al., 2018) and evokes short-latency excitatory responses in periaqueductal gray neurons (Falkner et al., 2020). Excitatory postsynaptic responses are evoked in the striatum when stimulating thalamic terminals in mice obtained by crossing the Vglut2-ires-Cre with lox-channelrhodopsin (ChR2) mice (Johnson et al., 2020). The Vglut2-ire-Cre mouse was used to study the role of reticulospinal neurons in locomotor control (Capelli et al., 2017).

#### ZsGreen-lox Mouse

The Ai6 mouse has been widely used to label cells expressing the Cre-recombinase (Madisen et al., 2012). After exposure to Cre-recombinase, the floxed STOP cassette is removed, and this results in the expression of the ZsGreen fluorescent protein under the control of the CAG promoter. Cells display intense labeling with ZsGreen as demonstrated by us (Fougère et al., 2020) and others (Steinkellner et al., 2018, 2019). In our previous study, we compared ZsGreen-lox mouse (Cre-negative) and Vglut2-ZsGreen (Cre-positive) brain sections and confirmed that before the introduction of Cre-recombinase, only a very low baseline level of fluorescence was present in brain slices of homozygous ZsGreen-lox mice (Fougère et al., 2020). This is classical for reporter lines based on CAG promoter-driven expression (e.g., Ai9, tdTomato-lox mouse) as mentioned by the supplier (Jackson laboratory).

#### ChR2-EYFP-lox Mouse

The Ai32 mouse (Madisen et al., 2012) has been widely used to activate cells expressing the Cre-recombinase using optogenetics (e.g., Caggiano et al., 2018; Josset et al., 2018). When exposed to Cre-recombinase, the floxed STOP cassette is removed, and this results in the expression of the ChR2(H134R)-EYFP fusion protein under the control of the CAG promoter.

### Statistical Analysis

Data are presented as mean ± standard error of the mean (SEM) unless stated otherwise. Statistical analyses were done using Sigma Plot 12.0. Normality was assessed using the Shapiro–Wilk test. Equal variance was assessed using the Levene test. Parametric analyses were used when assumptions for normality and equal variance were respected, otherwise, non-parametric analyses were used. To compare the means between two dependent groups, a two-tailed paired *t*-test was used. For more than two dependent groups, a parametric one-way analysis of variance (ANOVA) for repeated measures or a non-parametric Friedman ANOVA for repeated measures on ranks was used. ANOVAs were followed by a Student Newman-Keuls *post hoc* test for multiple comparisons between groups. Linear and nonlinear (sigmoidal) regressions between variables, their significance, and the 95% confidence intervals were calculated using Sigma Plot 12.0 Statistical differences were assumed to be significant when *P* < 0.05.

## Results

We targeted glutamatergic cells that expressed Vglut2 in the CnF for optogenetic stimulation. We examined the presence of such cells by crossing mice expressing the Cre-recombinase under control of the *Vglut2* promoter (Vglut2-Cre mouse) with mice expressing a green fluorescent protein in a Cre-dependent manner (ZsGreen-lox mice; Figure 1A). In the offspring (Vglut2-ZsGreen mice), many cells were positive for ZsGreen in the CnF (*n* = 3 mice; Figures 1C–E). Most of these ZsGreen-positive cells were immunopositive for NeuN (67/78 cells, 85.9%, *n* = 3 mice; Figures 1F,G), consistent with our previous measurements in these mice (94.2%, Fougère et al., 2020). To stimulate these cells with blue light, we crossed Vglut2-Cre mice with mice expressing ChR2 in a Cre-dependent manner (ChR2-EYFP-lox; Figure 1B). Using patch-clamp recording in slices of the offspring (Vglut2-ChR2-EFYP mice), we validated that blue light elicited spiking at short latency in MLR neurons (*n* = 2 neurons from two mice; Figure 1H).

We then activated CnF neurons in freely moving Vglut2-ChR2-EYFP mice in an open-field arena (Figure 2A). We implanted an optic fiber 500 μm above the right CnF and verified the expression of ChR2-EYFP in the CnF (Figure 2C) and the implantation sites (*n* = 5 mice; Figures 2B–D). Optogenetic stimulation of the CnF with blue light increased locomotor speed as shown by single animal data (Figures 2E,G) and data pooled from five mice (Figure 2I). Statistical analysis confirmed that speed was increased during optogenetic stimulation (*P* < 0.01 vs. prestim, Student Newman-Keuls after a one-way ANOVA for repeated measures, *P* < 0.01) and decreased after the light was switched off (*P* < 0.01 vs. opto stim; Figure 2K). Replacing the 470 with a 589 nm laser did not increase locomotion as shown by single animal data (Figures 2F,H) and data pooled from four mice (*P* > 0.05 one-way ANOVA for repeated measures; Figures 2J,L).

Next, we compared spontaneous and optogenetic-evoked locomotion in a transparent linear corridor. We tracked the movements of each paw frame by frame using DeepLabCut (Mathis et al., 2018; Nath et al., 2019; Figure 3A). The footfall pattern was similar during spontaneous and optogenetic-evoked locomotion (Figures 3B,C). We normalized the cycle duration as a function of the left hindlimb movements and observed again similar gait diagrams during spontaneous and optogenetic-evoked locomotion as shown by single animal data (Figures 3D,E) and data pooled from four mice (Figures 3F,G). We noticed, however, that mice were stepping faster during optogenetic-evoked locomotion as the cycle duration was shorter (*P* < 0.05 vs. spontaneous, paired *t*-test, *n* = 4 animals; Figure 3H) while stride length did not differ (*P* > 0.05; Figure 3I). This was associated with a shorter stance duration (*P* < 0.01; Figure 3J), but no modification of swing duration (*P* > 0.05; Figure 3K), consistent with the specific modulation of stance duration when speed increases during natural locomotion (Herbin et al., 2007). Altogether, this indicated that optogenetic CnF stimulation evoked a normal footfall pattern in Vglut2-ChR2-EYFP mice.

We then compared the limb kinematics by tracking each hindlimb joint (iliac crest, hip, knee, ankle, MTP) and the toe tip using DeepLabCut (Figure 4A). The stick diagrams were similar during spontaneous and optogenetic-evoked locomotion (Figure 4B). We compared the angular variations of the hip, knee, ankle, and MTP joints as a function of time (Figure 4C) and cycle duration was normalized relative to MTP movements (Figure 4D). The angular variations were similar during spontaneous and optogenetic-evoked locomotion as shown by single animal data and data pooled from four mice (Figure 4E). Statistical analysis revealed no difference in the angle amplitude of the four joints between the two conditions (*P* > 0.05 vs. spontaneous, paired *t*-test, *n* = 4 animals; Figure 4F). Altogether, this indicated that optogenetic CnF stimulation evoked normal limb kinematics.

We then examined whether freely behaving mice could brake or turn during optogenetic CnF stimulation in the open-field arena. Inspection of the speed as a function of time uncovered oscillations during optogenetic stimulation (Figures 5A,B). We plotted the speed as a function of the location of the animal in the arena and found that speed decreased in the corners of the arena, where the animal was performing turning movements (Figure 5C). This suggested that during CnF stimulation, the animal dynamically controlled speed as a function of environmental cues. We further studied this phenomenon by analyzing locomotor movements in each corner of the arena during CnF stimulation (Figure 5D). We defined ROIs as circles (20 cm radius) centered on each corner. The trajectories of a single mouse within the four ROIs during CnF stimulation are illustrated in Figure 5E. We defined the turning point as the intersection between the mouse’s trajectory and the corner’s bisector (i.e., arena’s diagonal). Plotting the speed relative to the distance from the turning point indicated that speed was lower around the turning point in single animal data (Figure 5G) as in data pooled from five mice (Figures 5F,H). Statistical analysis showed that speed decreased by ~61% during the turn (*P* < 0.05 vs. entry speed, Student Newman-Keuls after a one-way ANOVA for repeated measures, *P* < 0.01; Figure 5I). Speed increased when exiting the corner (*P* < 0.01 vs. turn speed; Figure 5I) to values that were not different from the entry speed (*P* > 0.05 vs. entry speed; Figure 5I). This indicated that the slowdown was transient and linked to the turn, after which ongoing MLR stimulation regained control over speed.

We examined the relationships between speed, braking, and turn angle. We found a strong positive linear relationship between the entry speed, and the speed decrease between entry and turning zone in five mice (*P* < 0.0001, *R* = 0.90; Figure 5J), and a strong positive linear relationship between the exit speed, and the speed increase between turning zone and exit (*P* < 0.0001, *R* = 0.92; Figure 5K). We found a weak but significant positive linear relationship between entry speed and turn speed (*P* < 0.05, *R* = 0.27; Figure 5L) and a weak but significant positive linear relationship between turn speed and turn angle (*P* < 0.0001, *R* = 0.44; Figure 5M). This last relationship is visible when looking at the trajectories color-coded as a function of turn speed (Figures 5E,F). This indicated that during CnF stimulation, a mouse running at high speeds was less able to make sharp turns, as reported during natural locomotion in mammals (Wynn et al., 2015).

**Figure 6:**
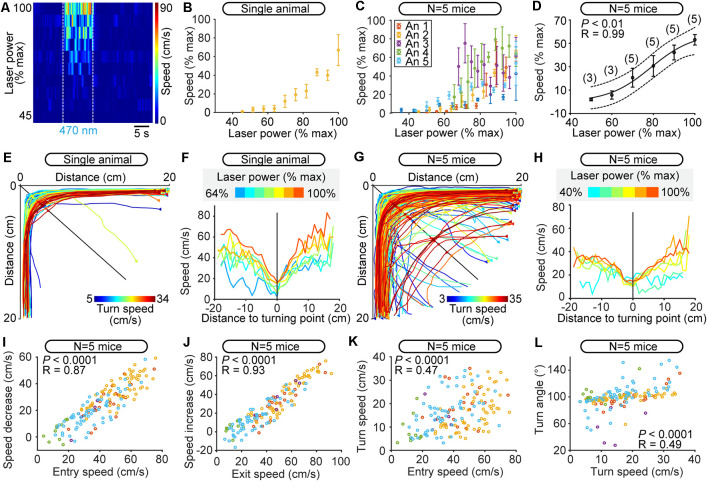
Robustness of scaled control of speed during turning at different speeds controlled by the level of optogenetic stimulation of the cuneiform nucleus (CnF) in Vglut2-ChR2-EYFP mice in the open-field arena. **(A)** Color plot illustrating the increase in overall locomotor speed in the open-field arena (cm/s) evoked by increases in laser power (6–13% of laser power) in a single animal (470 nm laser, 10 s train, 20 Hz, 10 ms pulses). Speed was calculated during the 10 s stimulation period, during which multiple turns could occur. Plotted laser powers were normalized as a percentage of their maximal value used per animal (% max). Each line illustrates the speed as a function of time for a given laser power expressed as a percentage of maximal laser power used for this animal. White dotted lines indicate the onset and offset of optogenetic stimulation. Warmer colors (red) indicate higher speeds. **(B)** Locomotor speed (1–42 cm/s) as a function of laser power (6–13% of laser power) in one animal. Each dot represents the speed (mean ± SEM) over three stimulations. Speed and laser power were normalized as a function of their maximal values (% max). **(C)** Relationship between locomotor speed (0.2–42.0 cm/s) and increasing laser power (6–27% of laser power) for all animals. Data from each mouse are illustrated with a different color. Each dot represents the speed (mean ± SEM) over three stimulations. Speed and laser power were normalized as a percentage of their maximal values per animal (% max). **(D)** Relationship between locomotor speed (mean ± SEM) and laser power in the same animals as in **C**, this time with data binned as a function of maximal laser power used per animal (% max) with a bin size of 10%. Speed and laser power were normalized as a percentage of their maximal values per animal (% max). The data followed a sigmoidal function (solid black line, *P* < 0.01, *R* = 0.99). The dotted lines illustrate the 95% prediction intervals. **(E–G)** Raw data showing the extracted locomotor trajectories in the corners of the arena during optogenetic-evoked locomotion in a single animal **(E)** and in five animals **(G)** Triangles illustrate movement onsets. Dots illustrate diagonal crossings. Warmer colors (red) illustrate higher turn speeds. **(F–H)** Locomotor speed as a function of the distance to the diagonal during the turns for increasing power of optogenetic stimulation of the CnF in a single animal (**F**, 470 nm laser, 10 s train, 20 Hz, 10 ms pulses, 6–13% of laser power) and in five animals (**H**, 470 nm laser, 10 s train, 20 Hz, 10 ms pulses, 6–27% of laser power). Warmer colors (red) indicate stronger optogenetic stimulation of the CnF. Laser powers were normalized as a percentage of their maximal value used per animal (% max) and were binned in **H** (bin width: 10%). In **(F)**, each curve was obtained from 1 to 14 turns in a single animal. In **(H)**, each curve was obtained from 2 to 73 turns pooled from five animals. **(I)** Relationship between the speed at corner entry, and the difference between entry speed and turn speed (linear fit, *P* < 0.0001, *R* = 0.87, *N* = 157 turns pooled from 150 stimulations, 30 stimulations per animal). **(J)** Relationship between the speed at corner exit, and the difference between exit speed and turn speed (linear fit, *P* < 0.0001, *R* = 0.93). **(K)** Relationship between the speed at corner entry and the turn speed (linear fit, *P* < 0.0001, *R* = 0.47). **(L)** Relationship between the turn speed and the turn angle (linear fit, *P* < 0.0001, *R* = 0.49).

We then examined the robustness of such scaled control of speed when making turns at different CnF stimulation strengths. We first determined whether CnF stimulation controlled the overall locomotor speed in the open-field arena. Note that multiple turns could occur during the 10 s of stimulation. Increasing the laser power applied to the CnF increased overall locomotor speed in the open-field arena as shown by single animal data (Figures 6A,B) and data pooled from five mice (Figure 6C). The minor differences in the relationship between laser power and speed from one animal to another (Figure 6C) might be related to the different depths of optic fiber tip position, and/or the different antero-posterior positions of the optic fiber (Figure 2D). We expressed the laser power and speed as a function of their maximal values per animal, and we found a strong positive sigmoidal relationship between laser power and speed (*P* < 0.01, *R* = 0.99; Figure 6D). Such precise control of speed confirmed that we successfully targeted the CnF (Figure 2D). Mice made to walk at increasing speeds imposed by increasing CnF stimulation were able to maintain successful braking and turning as shown by single animal data (Figures 6E,F) and data pooled from five mice (Figures 6G,H). The relationships describing the scaling of speed relative to the turn properties were conserved within this range of speeds (Figures 6I–L). This indicated that CnF stimulation controls locomotor speed, without preventing the animal from precisely regulating braking and turning, likely through the dynamic integration of environmental cues.

## Discussion

We show in Vglut2-ChR2-EYFP mice that optogenetic stimulation of the CnF with blue light evoked locomotion and that increasing laser power increased speed. Replacing the blue laser with a red laser evoked no locomotion. In a linear corridor, footfall patterns and limb kinematics were largely similar during spontaneous and optogenetic-evoked locomotion. In the open-field arena, mice could brake and perform sharp turns (~90°) when approaching a corner during CnF stimulation. Speed decrease during the turn was scaled to speed before the turn, and turn speed was scaled to turn angle. We verified the stimulation sites in the CnF and showed that most Vglut2-ZsGreen cells in the CnF were positive for NeuN. Using patch-clamp recordings in brainstem slices we showed that blue light evoked short-latency spiking. Altogether, our study indicates that the CnF controls locomotor speed without preventing the animal from integrating environmental cues to perform braking and turning movements, and thereby smoothly navigate the environment.

### Limits of the Study

We cannot exclude that some non-glutamatergic neurons were stimulated in the CnF of Vglut2-ChR2-EYFP mice. We crossed Vglut2-Cre with ChR2-EYFP-lox mice. In the offspring (Vglut2-ChR2-EYFP), if Vglut2 is expressed during cell lifetime, ChR2 is expressed permanently under the control of the CAG promoter even if Vglut2 is not expressed anymore (Steinkellner et al., 2018). We found that 85.9% of CnF ZsGreen-positive cells were NeuN-positive, consistent with our previous observations in the same mice (94.2%, Fougère et al., 2020). NeuN-negative cells may be cells for which NeuN labeling was too faint, glia (unlikely since astrocytes do not express Vglut2, see Li et al., 2013), or NeuN-negative neurons as observed in the cerebellum, olfactory bulb, retina, and substantia nigra pars reticulata (Mullen et al., 1992; Kumar and Buckmaster, 2007). We cannot rule out that our stimulations might have recruited Vglut2-positive neurons in neighboring regions such as the periaqueductal gray or the pedunculopontine nucleus, or fibers of passage. Future studies should use c-fos to anatomically identify the activated neurons. Mainly glutamatergic and GABAergic neurons are present in the CnF (Caggiano et al., 2018; Josset et al., 2018; for review Ryczko and Dubuc, 2013). Although the expression of Vglut2 was detected in some GABAergic neurons in the mammalian brain (Root et al., 2018), it is unlikely that we stimulated GABAergic neurons. We did not test the presence of *Vglut2* mRNA in ZsGreen positive cells in the CnF. However, in the offspring of Vglut2-Cre mice crossed with lox-GFP reporter mice, GFP-positive neurons are found in regions positive for the *Vglut2* mRNA and negative for the *vesicular GABAergic transporter* mRNA (Vong et al., 2011; see “Materials and Methods” section). Two arguments indicating that we successfully targeted CnF glutamatergic neurons are the normal gait diagrams and limb kinematics, and the precise control of speed when increasing laser power. These effects are consistent with results obtained in Vglut2-Cre mice optogenetically stimulated in the CnF following injection of an AAV encoding for ChR2 in a Cre-dependent manner (Caggiano et al., 2018; Josset et al., 2018). Such a virus-based approach that allows for more precise targeting of structures should be used in future studies to confirm the present results. Whether pooling the data from males and females might have contributed to the variability of the present results remains to be explored.

### Brainstem Control of Speed

Our results support the idea that MLR glutamatergic neurons play a key role in the initiation of forward symmetrical locomotion and in the control of speed by sending input to reticulospinal neurons (lamprey: Sirota et al., 2000; Brocard et al., 2010; salamander: Cabelguen et al., 2003; Ryczko et al., 2016a; mouse: Bretzner and Brownstone, 2013; Lee et al., 2014; Roseberry et al., 2016; Capelli et al., 2017; Caggiano et al., 2018; Josset et al., 2018) that send input to excitatory neurons of the locomotor central pattern generator (lamprey: Buchanan and Grillner, 1987; zebrafish: Kinkhabwala et al., 2011; Kimura et al., 2013; mouse: Hägglund et al., 2010; Capelli et al., 2017; salamander: Ryczko et al., 2020b). Here, we show that MLR stimulation does not prevent mice from braking and turning following the integration of environmental cues. The turning and braking movements recorded here displayed the same characteristics as those shown by mammals during natural locomotion. At high speed, mice used fewer sharp turns (i.e., higher angles), consistent with observations in wild northern quolls, which reduce their locomotor speed more during turns with a smaller radius (Wynn et al., 2015).

### Brainstem Control of Braking and Turning

**Figure 7:**
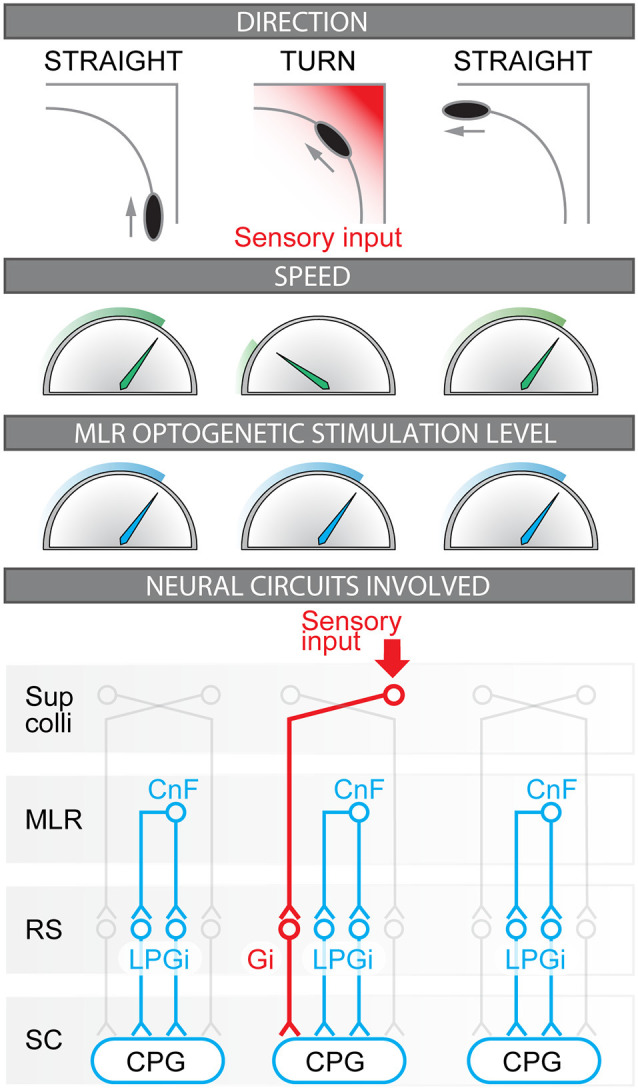
Coordinated control of locomotor speed and turning movements during optogenetic stimulation of the Mesencephalic Locomotor Region (MLR). Illustration of the relation between motion direction, locomotor speed, and MLR optogenetic stimulation level in the present study. The neural circuits likely involved before, during, and after the turn are illustrated. Before the turn, forward locomotion is evoked by unilateral stimulation of glutamatergic neurons of the cuneiform nucleus (CnF, part of the MLR) that provides bilateral activation of reticulospinal (RS) neurons located in the lateral paragigantocellular nucleus (LPGi) that project to the spinal neurons of the central pattern generator (CPG) for locomotion (mouse Bretzner and Brownstone, 2013; Lee et al., 2014; Roseberry et al., 2016; Capelli et al., 2017; Josset et al., 2018; Caggiano et al., 2018). Also see corresponding studies in lamprey (Buchanan and Grillner, 1987; Brocard and Dubuc, 2003; Le Ray et al., 2003; Brocard et al., 2010), zebrafish (Kinkhabwala et al., 2011; Kimura et al., 2013), and salamander (Cabelguen et al., 2003; Ryczko et al., 2016a). During the turn, the visual inputs conveying the approach of the corner are relayed by the superior colliculus (Sup Colli) that sends projections to contralateral reticulospinal neurons of the gigantocellularis nucleus (Gi) that evoke ipsilateral braking and turning movements (Bouvier et al., 2015; Cregg et al., 2020; Usseglio et al., 2020); also see studies on the role of reticulospinal neurons in steering control in lamprey (Deliagina et al., 2000; Fagerstedt et al., 2001; Kozlov et al., 2014; Suzuki et al., 2019), zebrafish (Huang et al., 2013; Thiele et al., 2014), salamander (Ryczko et al., 2016c) and rat (Oueghlani et al., 2018). After the turn, the sensory inputs generated by the corner disappear, Gi neurons are deactivated, speed increases back to the value set by the steady MLR command, and forward symmetrical locomotion is restored.

Our observations support the idea that distinct brainstem neurons control speed and turning/braking movements. Our data indicate that a substrate for turning movements is activated transiently during MLR stimulation when approaching a corner. This motor signature closely matches that previously recorded when selectively activating the brainstem circuit for turning (Cregg et al., 2020; Usseglio et al., 2020). In mice, a bilateral activation of Gi Chx10-positive neurons evokes a locomotor arrest *in vivo* (Bouvier et al., 2015) whereas a unilateral activation produces an ipsilateral turn (Cregg et al., 2020). The reticulospinal nature of the neurons involved was demonstrated *in vivo* using unilateral optogenetic stimulation in the Gi of Chx10-positive neurons retrogradely labeled by viral injections at different levels of the spinal cord (Usseglio et al., 2020). The lumbar-projecting Gi Chx10-positive neurons decrease locomotor speed, whereas the cervical-projecting ones produce the ipsilateral turn (Usseglio et al., 2020). Gi-Chx10 neurons receive a major input from the contralateral superior colliculus (SC), a region involved in visuomotor transformations (Cregg et al., 2020; see also Liang et al., 2015). Such connectivity is relevant to the behavioral task mice had to solve here in the open-field arena, i.e., integrating visual cues to avoid the arena’s corner during locomotion evoked by MLR stimulation (Figure 7). Altogether, this suggests that the brainstem substrates for braking and turning (Bouvier et al., 2015; Cregg et al., 2020; Usseglio et al., 2020) can be recruited during MLR stimulation, therefore allowing the animal to smoothly navigate the environment (Figure 7).

Interestingly, the speed decreased to zero during some turns, i.e., mice transiently halted during optogenetic MLR stimulation (Figure 5G). Future studies should examine which level of locomotor circuitry is involved in this effect. At the reticulospinal level, “stop cells” could increase their activity to stop locomotion as shown in basal vertebrates (lamprey: Juvin et al., 2016; Grätsch et al., 2019). In mammals, a halt is induced by bilateral recruitment of Gi Chx10-positive neurons, i.e., the same neurons that induce turning when activated unilaterally (Bouvier et al., 2015; Cregg et al., 2020; see also Liang et al., 2015). Interestingly, a recent study uncovered that unilateral stimulation of reticulospinal Gi Chx10-positive neurons projecting to the lumbar spinal cord decreases locomotor speed without inducing turning (Usseglio et al., 2020). *In vivo* calcium imaging showed that Gi Chx10-positive neuron activity increases during locomotor stops in mice (Schwenkgrub et al., 2020). Locomotor pauses and rhythm resetting were also reported when photoactivating Vglut2-positive neurons in the Gi *in vivo* in mice (Lemieux and Bretzner, 2019). Neurons positive for the glycinergic transporter 2 (Glyt2) in the LPGi, Gi, GiA, and GiV were found to evoke different forms of locomotor arrests *in vivo* (Capelli et al., 2017). At the MLR level, local GABAergic neurons could stop locomotion likely by inhibiting MLR glutamatergic neurons (Roseberry et al., 2016; Caggiano et al., 2018). In lamprey, stimulation of the MLR, at lower stimulation intensity values than the ones evoking locomotion, stops locomotion by recruiting reticulospinal stop cells (Grätsch et al., 2019). Two incoming inputs to the MLR could be involved. A transient increase in the GABAergic tone from the output stations of the basal ganglia could stop locomotion (lamprey: Ménard et al., 2007; Stephenson-Jones et al., 2011; mouse: Kravitz et al., 2010; Roseberry et al., 2016). Alternatively, increased activity from the output station of the basolateral amygdala could be involved, since activation of this region is synchronized with locomotor arrests in familiar places during exploratory behavior (Botta et al., 2019; for review Roseberry and Kreitzer, 2017).

The open field provides a relatively modest challenge of avoiding walls when turning. Future studies should examine whether mice receiving MLR stimulation can smoothly perform in navigation tests of higher complexity with interconnected narrow corridors, such as the radial arm or the complex maze tests. Whether clearance of obstacles located in the middle of the path of a mouse performing MLR-evoked high-speed locomotion is possible should also be examined. To identify the limb pattern during turns, future studies should use an open-field arena with a glass floor and film the animals from below during MLR stimulation.

## Conclusions

We show that optogenetic stimulation of the CnF in Vglut2-ChR2-EYFP mice controls locomotor speed without preventing braking and turning movements following the integration of environmental cues. This supports the idea that distinct brainstem circuits control speed (Lee et al., 2014; Roseberry et al., 2016; Capelli et al., 2017; Caggiano et al., 2018; Josset et al., 2018) and braking/turning movements in mammals (Bouvier et al., 2015; Lemieux and Bretzner, 2019; Cregg et al., 2020; Usseglio et al., 2020; Figure 7). This also suggests that MLR glutamatergic neurons (and especially CnF glutamatergic neurons, Chang et al., 2020) are a relevant target to improve navigation adaptable to the environment in conditions where locomotion is impaired such as Parkinson’s disease (Plaha and Gill, 2005; Hamani et al., 2016a, b; Goetz et al., 2019), spinal cord injury (Bachmann et al., 2013; Richardson, 2014; Roussel et al., 2019; for review Chari et al., 2017) and stroke (Fluri et al., 2017).

## Data Availability Statement

The raw data supporting the conclusions of this article will be made available by the authors, without undue reservation.

## Ethics Statement

The animal study was reviewed and approved by the animal care and use committees of the Université de Sherbrooke.

## Author Contributions

CIvdZ: conceptualization, data curation, formal analysis, investigation, methodology software, validation, visualization, roles/writing—original draft, writing—review and editing. JB, MF and AF: data curation, investigation, methodology, validation, visualization, writing—review and editing. MV: methodology, writing—review and editing. AS, TA and PS: methodology, software, writing—review and editing. DR: conceptualization, data curation, formal analysis, funding acquisition, methodology, project administration, resources, supervision, validation, visualization, roles/writing—original draft, writing—review and editing. All authors contributed to the article and approved the submitted version.

## Conflict of Interest

The authors declare that the research was conducted in the absence of any commercial or financial relationships that could be construed as a potential conflict of interest.
